# Natural Mineral Particles Are Cytotoxic to Rainbow Trout Gill Epithelial Cells *In Vitro*

**DOI:** 10.1371/journal.pone.0100856

**Published:** 2014-07-03

**Authors:** Christian Michel, Simon Herzog, Christian de Capitani, Patricia Burkhardt-Holm, Constanze Pietsch

**Affiliations:** 1 Man-Society-Environment (Programm MGU), Department of Environmental Sciences, University of Basel, Basel, Switzerland; 2 Mineralogy-Petrography, Department of Environmental Sciences, University of Basel, Basel, Switzerland; 3 Department of Biological Sciences, University of Alberta, Edmonton, Canada; University of Windsor, Canada

## Abstract

Worldwide increases in fluvial fine sediment are a threat to aquatic animal health. Fluvial fine sediment is always a mixture of particles whose mineralogical composition differs depending on the sediment source and catchment area geology. Nonetheless, whether particle impact in aquatic organisms differs between mineral species remains to be investigated. This study applied an *in vitro* approach to evaluate cytotoxicity and uptake of four common fluvial mineral particles (quartz, feldspar, mica, and kaolin; concentrations: 10, 50, 250 mg L^−1^) in the rainbow trout epithelial gill cell line RTgill-W1. Cells were exposed for 24, 48, 72, and 96 h. Cytotoxicity assays for cell membrane integrity (propidium iodide assay), oxidative stress (H_2_DCF-DA assay), and metabolic activity (MTT assay) were applied. These assays were complemented with cell counts and transmission electron microscopy. Regardless of mineral species, particles ≤2 µm in diameter were taken up by the cells, suggesting that particles of all mineral species came into contact and interacted with the cells. Not all particles, however, caused strong cytotoxicity: Among all assays the tectosilicates quartz and feldspar caused sporadic maximum changes of 0.8–1.2-fold compared to controls. In contrast, cytotoxicity of the clay particles was distinctly stronger and even differed between the two particle types: mica induced concentration-dependent increases in free radicals, with consistent 1.6–1.8-fold-changes at the 250 mg L^−1^ concentration, and a dilated endoplasmic reticulum. Kaolin caused concentration-dependent increases in cell membrane damage, with consistent 1.3–1.6-fold increases at the 250 mg L^−1^ concentration. All effects occurred in the presence or absence of 10% fetal bovine serum. Cell numbers *per se* were marginally affected. Results indicate that (*i*.) natural mineral particles can be cytotoxic to gill epithelial cells, (*ii*.) their cytotoxic potential differs between mineral species, with clay particles being more cytotoxic, and (*iii*.) some clays might induce effects comparable to engineered nanoparticles.

## Introduction

Suspended mineral particles are a natural and ubiquitous component of the aquatic environment [1]. Freshwater fish have successfully evolved in this particle-rich environment [2]. However, anthropogenic fine sediment input is also increasing in many rivers worldwide [3]–[5]. Especially in high-altitude areas of the Northern Hemisphere, climate change will likely further promote this trend [6]–[8]. These observed and predicted increases of suspended fluvial mineral particles have raised concerns about negative effects on aquatic biota, including freshwater fish [2], [5], [9]–[11].

The fish gill was often considered a primary target organ for suspended mineral particle effects [5]. Yet, especially for the smaller particle sizes of silt (≤63 µm) and clay (≤2 µm), evidence about gill epithelial damage is inconclusive: Some studies reported structural gill damage after exposure (270–4887 mg L^−1^, [12], [13], [14]), whereas other studies did not find effects using comparable particle sizes, concentrations and exposure durations [15]–[17]. One possible explanation is differences in the geochemical composition of the tested particles. For example, it has been suggested that clay particles may have a stronger impact because of their smaller size and higher surface reactivities, leading to higher reactivity towards cellular structures [5], [18]. Small-sized clay particle pulses (mica/muscovite, <30 µm, 300 mg L^−1^ max. concentration) were recently found to cause slight lipid peroxidation, but no structural damage, in the gill of rainbow trout [16]. Together, these data suggest that (*i*.) cytotoxic effects might differ between different mineral species and (*ii*.) that silt- and clay-sized mineral particles might be particularly cytotoxic to gill epithelial cells. However, while there is some evidence for this it has, to our knowledge, not yet been experimentally investigated.

Aquatic organisms are regularly exposed to natural mineral particles (“NMPs”) in the low µm to nm size-range [19], [20], i.e. sizes comparable to engineered nanoparticles (“ENPs”; [21], [22]). ENPs are defined as particles with at least one dimension below 100 nm [23], [24]. The size of ENP agglomerates in water range from hundreds of nanometers to a few micrometers [25]–[27], and ENP agglomerates need to be considered in ecotoxicological risk assessment [28], [29]. Interestingly, the amount of NMPs in aquatic ecosystems probably by far outweighs the amount of anthropogenic ENPs released to the environment [19], [21]. In eco-toxicological risk assessments, this raises the question whether NMPs induce comparable cytotoxic effects to ENPs [21], [22].

Many of the properties considered important for cytotoxic effects of NMPs [30] and ENPs [29] are comparable. For both, particle size and shape jointly determine the surface area of a particle sample, and hence are important for cellular effects to develop [30]–[32]. The cytotoxic potential usually increases for samples with smaller particle sizes because they have a larger surface area per unit mass (“specific surface area”, [32]). The biological activity of the particles is also related to functional surface groups, which affect particle surface charges and reactivity [33]–[35]. Toxic compounds bound to particle surfaces can further contribute to their toxicological potential [33], [36]. While these aspects indicate that the cytotoxicity of NMPs and ENPs could be comparable [19], the novel tailor-made surface properties of some ENPs might induce different cytotoxic pathways than NMPs [37]. Properties of some ENPs, absent in NMPs, include specific surface chemistries (e.g. citrate, graphene cage arrangements of carbon atoms), unnatural incidences of a single surface ligand (NMPs mostly have a mixture of ligands), and specific particle shapes rarely found in NMPs (e.g. rod shaped, rosettes). ENP cytotoxicity has been investigated in salmonid gill epithelial cells (i.e. RTgill-W1 cell line; [38], [39], [40]). To date, however, no data are available for natural silt- and clay-sized mineral particles.

The current study applied an *in vitro* approach to test specific questions about the cytotoxicity of mineral particles to gill epithelial cells of a salmonid fish. For this, the rainbow trout epithelial gill cell line RTgill-W1 [41] was used to investigate (*i*.) whether small-sized mineral particles cause cytotoxic and/or ultrastructural effects in gill epithelial cells, and (*ii*.) whether the particles are taken up by the cells. These data were complemented with cell counts to distinguish effects related to changes in cell numbers from changes in toxicity end-points per cell. Further, changes in cell numbers could also indicate particle-induced cell death. Finally, we investigated whether the type of effect varied between exposure conditions and different mineral species. To investigate mineral species effects, the four silicate minerals quartz, feldspar, mica, and kaolin, which are common in European and North American watersheds [42]–[45], were selected. These particles represent the two bulk structures of tectosilicates (quartz, feldspar; “framework silicates”) and phyllosilicates (mica, kaolin; “clays”). These clay particles were specifically included because it has been suggested that the higher surface reactivity of clay particles cause stronger effects in aquatic organisms [5], [18]. The results presented here provide first empirical data on cytotoxic effects of natural silt- and clay-sized mineral particles to salmonid gill epithelial cells. Thus, they provide new insights for the eco-toxicological risk assessment of suspended NMPs, whose occurrence is predicted to increase in many rivers due to altered land-use practices and climate change (e.g., [6], [8], [10]).

## Materials and Methods

### Preparation and characterization of particles

Commercial mineral particles (quartz (silica), potassium feldspar (orthoclase), mica (muscovite), and kaolin (kaolinite)) were used because of their purity and defined mineral composition. Particle sizes (Table 1) were selected to be similar to particles transported in rivers [44], and to comprise the size range of natural colloids (i.e., ≤1 µm; [20]) and ENP agglomerates in water (i.e., ≤3 µm; [25], [26], [27]). The size distribution of each particle sample was quantified directly in the cell culture medium also used in the experiments described below (i.e. Leibovitz's L-15 medium (LuBio Science GmbH, Switzerland) containing 100 U mL^−1^ penicillin and 100 µg mL^−1^ streptomycin (“pen/strep”) and supplemented once with and once without 10% fetal bovine serum (“FBS”, Sigma-Aldrich GmbH, Switzerland). Measurements were conducted in an Ultrasizer X particle size analyzer (Malvern Instruments Ltd., United Kingdom) with each particle sample measured in triplicate. For the measurements, 70 mL of the respective exposure medium was added to the dispersion unit (Hydro 2000SM, Malvern Instruments Ltd., United Kingdom) to equilibrate the measurement device. In parallel, the particle sample was suspended in 50 mL exposure medium and dispersed twice for 1 min by ultra-sonication (35 kHz, Sonorex, Bandelin GmbH, Germany). Then the background obscuration of the exposure medium was measured. Once the background obscuration reached 0%, the particle suspension was added to the dispersion unit, and the particle size distribution was quantified. According to manufacturer's data the specific surface area of the clay samples was 21 m^2^ g^−1^ (mica) and 11 m^2^ g^−1^ (kaolin). For the framework silicate samples, no data were available. Yet, quartz particle samples from the same supplier (D_95_<8.2 µm; Note: D_95_ = 95% quantile of the particle sizes in a sample) had a specific surface area of 4.2 m^2^ g^−1^ [46]. For feldspar samples of other origin (D_95_<20 µm) a specific surface area of 3.6 m^2^ g^−1^ was reported [47]. The specific surface area of mineral particle samples is negatively related to particle size [48]. These data indicate that the clay samples (D_90_<22 µm) had at least a two-fold higher specific surface area compared to the framework silicates (D_90_<40 µm). Prior to use in the experiments, the particles were pre-weighed in 1.5 mL reaction tubes and sterilized by γ irradiation (100 Gy, 42 h; Gammacell 40 Extactor, Theratronics Inc., Canada). This is a common method to sterilize nanoparticles and has minimal effects on particle properties [49]. Mineralogy and purity of the particle samples were determined before and after gamma irradiation using x-ray diffractometry (Diffractometer D5000, Siemens, Germany). The mineralogical composition of each particle sample was then approximated by comparing peak intensities and correcting for known ratios relative to corundum with the software program Diffrac Plus EVA v.13.0.0.2 (Bruker-AXS Inc., U.S.A.; e.g., [50]).

**Table 1:** Particle size characterization of the investigated particle samples.

Mineral	FBS	D_10_	D_50_	D_90_
Quartz	Yes	1.90±0.02	13.49±0.09	37.20±0.23
	No	1.84±0.01	12.98±0.15	39.73±0.20
Feldspar	Yes	1.79±0.01	11.86±0.15	35.57±1.42
	No	1.88±0.03	10.97±0.41	34.27±1.07
Mica	Yes	1.43±0.01	4.24±0.01	10.41±0.05
	No	1.45±0.07	3.96±0.01	7.44±0.10
Kaolin	Yes	2.11±0.03	6.66±0.03	21.95±0.73
	No	1.98±0.01	6.60±0.07	20.31±0.80

Given are the 10% (D10), 50% (D50), and 90% (D90) quantiles of the respective mineral particle sample (Mineral) size distribution measured in L15 medium with pen/strep and once with and once without 10% FBS. Data in micrometers (µm) given as mean ± SEM (*n* = 3).

### Cell culture

The rainbow trout (*Oncorhynchus mykiss*) epithelial gill cell line RTgill-W1 [41], kindly provided by Kristin Schirmer (EAWAG, Dübendorf, Switzerland), was used for the experiments. Cells were cultivated in 75 cm^2^ cell-culture flasks (material: polystyrol without surface modification, TPP AG, Switzerland) using L-15 medium with pen/strep containing 10% FBS. Cells were maintained at 19°C in normal atmosphere (ambient gas composition: 21% O_2_, 78% N_2_, and 0.04% CO_2_) with no CO_2_ supplementation and sub-cultured weekly. For sub-culturing and seeding in plates for cytotoxicity assessments (see below), cells were harvested by flushing with 2.5 ml versene (LuBio Science) twice followed by incubation with trypsin (BioSera, Socochim SA, Lausanne, Switzerland) for 3 min. Trypsin treatment was terminated by adding 5 ml of L-15 medium containing 10% FBS. The obtained cell suspensions were centrifuged at 1,000 rpm for 5 min (Megafuge 1.0R, Heraeus), re-suspended in medium, and cells were enumerated using a Neubauer hemocytometer cell counting chamber.

### Cytotoxicity assessment

For cytotoxicity assessment, cells were seeded in L-15 medium (containing pen/strep and 10% FBS) in 96-well cell culture plates (material: polystyrol without surface modification, TPP AG, Switzerland) at a concentration of 3.5×10^4^ cells in 200 µl medium per well. In the plates, cells were grown to confluence at 19°C in normal atmosphere for 48 h [51]. Then the medium was exchanged and cells were exposed to 200 µl particle working suspension (see below) for 24, 48, 72, and 96 h. All assays were conducted in L-15 medium with pen/strep once with and once without 10% FBS. These two FBS treatments were included because it is known from ENP research that FBS can coat particles, which could alter their interaction with cells and their cytotoxic potential (e.g., [39], [49]).

To obtain particle working suspensions, stock suspensions (1,250 mg L^−1^; 15–20 mL) were freshly prepared in L-15 medium before use in the experiments. Particles in the stock suspensions were dispersed twice by sonication for 1 min (35 kHz, Sonorex, Bandelin GmbH, Germany). Particle working suspensions of 10 mg L^−1^ (low), 50 mg L^−1^ (medium) and 250 mg L^−1^ (high) were then prepared by dilution from the stock suspension. On every plate blank wells containing only the respective exposure medium without cells and particles, and control wells containing only cells in the respective exposure medium were included. For each of three independent experimental blocks, one plate with eight wells per treatment level per time-point was used. Within each experimental block, treatments with and without FBS were included on the same plate, while, for technical reasons, the different mineral species and time-points were measured on separate plates. Thus, altogether a total of 16 plates were measured per experimental block.

For characterizing NMP cytotoxicity, three assays measuring distinct cellular endpoints were applied: Membrane integrity of individual cells was assessed using the intact cell membrane-impermeable fluorescent dye propidium iodide (PI assay). Upon cell membrane damage this dye enters the nucleus and binds to the DNA, which results in increased light emission upon excitation. Thus, increased signal intensities indicate impaired cell membrane integrity. Cell viability and metabolic activity were evaluated using the MTT assay [52]. This assay is based on the intracellular metabolic reduction of the cell membrane-permeable yellow thiazolyl blue tetrazolium bromide (MTT) salt to a purple insoluble formazan product in viable cells. Thus, in the MTT assay an increase in signal indicates either (*i*.) increased metabolic and mitochondrial activity of viable cells and/or (*ii*.) an increase in viable cell numbers [53]. Oxidative stress in the cells after particle exposure was quantified with the H_2_DCF-DA assay. Intracellular conversion of this cell membrane-permeable dye by free radicals causes an increased emission upon excitation. Thus, an increase in signal intensity indicates increased free radicals and hence oxidative stress. All applied cytotoxicity endpoints measure responses in cells; detailed descriptions of the applied methodology, reagents and instruments are given in Pietsch et al. [54]. One modification was introduced in the protocol: In the current study, each well was washed once with the buffer used in the respective dye solution before application of the indicator dye. This was done to reduce particle interference with the cytotoxicity assays (cf. [55]).

To assess whether the mineral particles *per se* affect the measurements, all cytotoxicity assays were also conducted using particle suspensions in the absence of cells. Particle suspensions of 10 mg L^−1^ (low), 50 mg L^−1^ (medium), and 250 mg L^−1^ (high concentration) were prepared as described above, and again once with and once without FBS. Suspensions were added to 96-well tissue culture plates (material: polystyrol without surface modification, TPP AG, Switzerland) with control wells (containing no particles) included on every plate. For each of three independent experiments, one plate each with three wells per mineral per treatment level was measured. Plates were incubated at 19°C in normal atmosphere for 72 h, and all assays were conducted as described above. All chemicals were obtained from Sigma-Aldrich (Switzerland).

To compare the relative cytotoxicity of the four mineral species studied, the following ranking procedure was applied for each cytotoxicity assay separately: First, the maximum fold-change in the 250 mg L^−1^ particle concentration was identified (Figures S1 and S2 in File S1). Then, the range spanning from the control ±10% to this maximum fold-change was divided into three equal intervals. (Note: To be conservative, only significant changes beyond ±10% from controls were considered biologically relevant in the ranking, since controls commonly spread in this range). The resulting fold-change intervals were then used to rank the effect size in three distinct categories (slight, moderate, strong) caused by the 250 mg L^−1^ concentration of each mineral type studied. The fold-change values attributed to each category can be found in Table S1 in File S1. This ranking procedure was conducted only for the cell membrane integrity and oxidative stress assays. The metabolic activity assay was not ranked because the particles *per se* regularly interfered with this assay to an extent that no safe conclusions could be drawn from the cell assays (see below).

### Cell numbers

To assess effects of the mineral particle exposure on cell numbers, RTgill-W1 cells in L-15 medium (pen/strep, with 10% FBS) were seeded in 24-well tissue culture plates (material: polystyrol without surface modification, Greiner BioOne, Germany) at a concentration of 1.4×10^5^ cells in 800 µl medium per well. Cells were grown to confluence at 19°C in normal atmosphere for 48 h [51]. The medium was then exchanged, and cells were exposed to a 250 mg L^−1^ mineral particle working suspension made with L-15 containing pen/strep, and once with and once without 10% FBS (prepared as described above). After 72 h, cell nuclei were stained with the cell membrane-permeable fluorescent stain Hoechst 33342 (5 µg mL^−1^ for 10 min; Molecular Probes, Invitrogen Inc.). Eight random photographs (400× magnification) were taken of each well along two central transects using a DMI 6000B inverted microscope and the LAS AF software v2.2.0 (both Leica Microsystems GmbH, Germany). Cell numbers were determined by counting cell nuclei in two random fields per picture using the Fiji distribution of ImageJ [56]. The counting order was randomized and sample names were blinded to avoid subjective bias. To complement these direct cell counts, changes of viable cell numbers in controls with and without FBS were approximated for each time-point using the metabolic activity in controls [53]. For each of three independent experimental blocks, one plate each with four wells per mineral per treatment level was counted.

### Particle uptake and cellular effects

Particle uptake and the ultrastructure of cells exposed to the particles were studied by transmission electron microscopy (TEM). For this, approximately 7.7×10^6^ cells in 12.5 ml L-15 medium with pen/strep, and once with and once without 10% FBS, were seeded into 75 cm^2^ culture flasks (material: polystyrol without surface modification, TPP AG, Switzerland) and grown to confluence for 48 h [51]. Then the medium was discarded and cells were exposed to 12.5 mL of a 250 mg L^−1^ mineral particle working suspension made with L-15 containing pen/strep, and again once with and once without 10% FBS (prepared as described above). Control cells were cultivated under exactly the same conditions but in particle-free medium. Cells were incubated for 72 h. After this time, adherent cells were washed twice, once with the respective particle-free medium (L-15, with pen/strep, either with or without 10% FBS) and once with FBS-free Earle's medium. Cells were fixed in 3% Karnofski paraformaldehyde containing 0.5% glutaraldehyde in phosphate-buffered saline for 1 h and then washed with PBS. Subsequently, cells were post-fixed in 1% osmium tetroxide containing 1.5% potassium ferrocyanide for 1.2 h and washed with water. Cells were dehydrated in an ascending ethanol series and acetone, stained with 6% uranyl acetate, and embedded in epoxy resin [57]. The resin was hardened for 48 h at 60°C and samples were sectioned at 60 nm thickness on a microtome (Jung Ultracut E, Reichert Microscope Services, USA) equipped with a diamond knife (Diatome AG, Switzerland). TEM examinations were made with a Morgagni 286(D) transmission electron microscope (FEI Company, USA). For semi-quantitative assessment of particle uptake and cyto-pathological effects, particle-exposed cells were screened in comparison to control cells. Cells showing obvious signs of particle uptake and/or cyto-pathological alterations were photographed. A total of 230 pictures were taken. Only effects observed in particle-exposed cells but absent in control cells are reported. The entire particle uptake experiment was replicated twice, always with particle-exposed cells and control cells processed in parallel. All TEM sample preparations and analyses were conducted at the Center for Microscopy and Image Analysis (ZMB) of the University of Basel. The detection limit for mineral particles in the cells was in the range of 20–30 nm (M. Dürrenberger, ZMB, University of Basel, pers. comm.).

### Statistical analysis

All statistical analyses were conducted using the open-source statistics software R v2.12.0 [58]. Significance was accepted at *p*≤0.05. Continuous response variables were analyzed using linear mixed effect models (function: lmer, lme4 package), with plates included as random factor to account for spatial clustering of wells (cf. [59]). The fixed effect part of the model consisted of the treatment level as categorical explanatory variable, and the fold-change (all time-points) or the raw response data (72 h time-point only) as continuous response variable. Significance was tested using likelihood ratio tests [59]. Once a significant main effect was detected, each treatment level was compared to the respective control using the Markov chain Monte Carlo (MCMC) re-sampling approach implemented in the function pvals.fnc (languageR package; [60]). Cell count data were analyzed using generalized linear mixed effect models (glmm) fitted with a Poisson error distribution and a log*_e_* link function (function: glmmPQL, MASS package; [61]). Fixed and random effect terms were included as described above. Significance was tested with Wald χ^2^ tests (function: wald.test, aod package). Once a significant main effect was detected, each particle treatment level was compared to the respective control using Wald *t*-tests [62]. If outliers were observed, the model was fitted with and without these data-points, and significance was accepted only when supported in both analyses. Model fit and assumptions were evaluated according to standard procedures [59], and no violations were observed. Finally, to test for changes in metabolic activity of the controls over time, the Pearson product-moment correlation analysis (function: cor.test) was applied.

## Results

### Particle characterization

The particle size distributions (Table 1) of the two clay particle samples (mica D_95_<11 µm;, kaolin D_90_<22 µm) were distinctly smaller than for the two framework silicate samples (quartz D_90_<40 µm; feldspar; D_90_<36 µm). The presence of 10% FBS in the medium had negligible effects on the particle size distributions (Table 1). X-ray diffractometry confirmed the mineralogical structure and purity of all mineral particle mixtures. Altogether, the mineralogical composition of the particle samples was as follows: quartz (≥98% pure quartz with ≤2% impurities), feldspar (∼90% feldspar, ∼10% quartz, with ≤2% impurities), mica (∼90% orthoclase, ∼10% quartz, ≤2% impurities) and kaolin (≥92% kaolinite, 5–8% quartz, with ≤3% impurities). In summary, all particle samples had ≥90% purity with some quartz (up to 10%) and less than 5% of other crystalline phases. No changes in mineral purity and crystallinity occurred after γ-irradiation.

### Particle uptake and cellular effects

**Figure 1:**
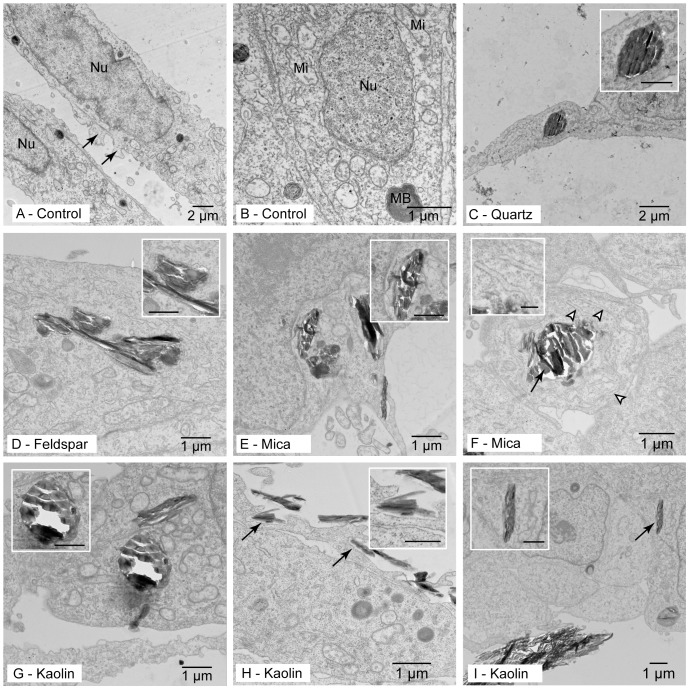
Control cells as well as particle uptake and effects. Pictures were taken after 72(control) or with 250 mg L^−1^ of the respective particle. Shown are examples for A.) Control cells, with marked nucleus (Nu) and arrows pointing to rudimentary microridges. B.) Control cell detail, with nucleus (Nu), mitochondria (Mi) and a myelin body (MB). C.) Quartz particle, phagocytized. D.) Feldspar particle, phagocytized. E.) Mica particle, phagocytized. F.) Mica particle, dilated endoplasmic reticulum. G.) Kaolin particle, phagocytized. H.) Kaolin particle phagocytosis (arrows, with inset showing detail of particle marked on the upper left). I.) Kaolin particle in cytoplasm without surrounding membrane. In all insets bar denotes 0.5 µm. Note: white areas around particles represent artifacts due to sectioning.

Ultrastructurally, the RTgill-W1 cells used here were very similar to those described by Bols and co-workers [41]. Irrespective of the presence of FBS, the control cells were elongated or spindle-shaped (Figure 1A). Mostly, cells were present in layers of two to three or showed overlapping cellular processes. Microridges, typical for gill epithelial cells in native epithelia, were irregularly shaped, but were rare (Figure 1A). Cells revealed a centrally located round to ellipsoid, slightly irregular-shaped nucleus. Patches of heterochromatin were distributed in the nucleus or attached to the nuclear membrane. Cell organelles were distributed throughout the cytoplasm, with a higher abundance near the nucleus (Figure 1B). In all cells, ribosomes were abundant, mostly near the endoplasmic reticulum (rough ER). Highly electron-dense lysosomes of up to 1 µm diameter were common; some showed layered membrane structures, which are most likely myelin bodies (Figure 1B). Microfilament bundles aligned with the elongated cells appeared regularly in the cytoplasm.

Regardless of the mineral particle studied and the presence of FBS, all cells appeared intact and without pronounced ultrastructural changes when compared to unexposed cells. All particles studied were found in membrane-bound vacuoles within the cytoplasm (Figure 1C–E and G). The size of intracellular particles ranged from 0.1 to 2 µm, and uptake occurred irrespective of the presence of FBS. In cells exposed to mica particles, a slightly dilated endoplasmic reticulum was noted (Figure 1F). Phagocytic uptake occurred (Figure 1H). Qualitatively, mica and kaolin particles were found in cells more often than quartz and feldspar. Kaolin particles were occasionally present without a surrounding membrane (Figure 1I).

### Cytotoxicity assays

**Figure 2:**
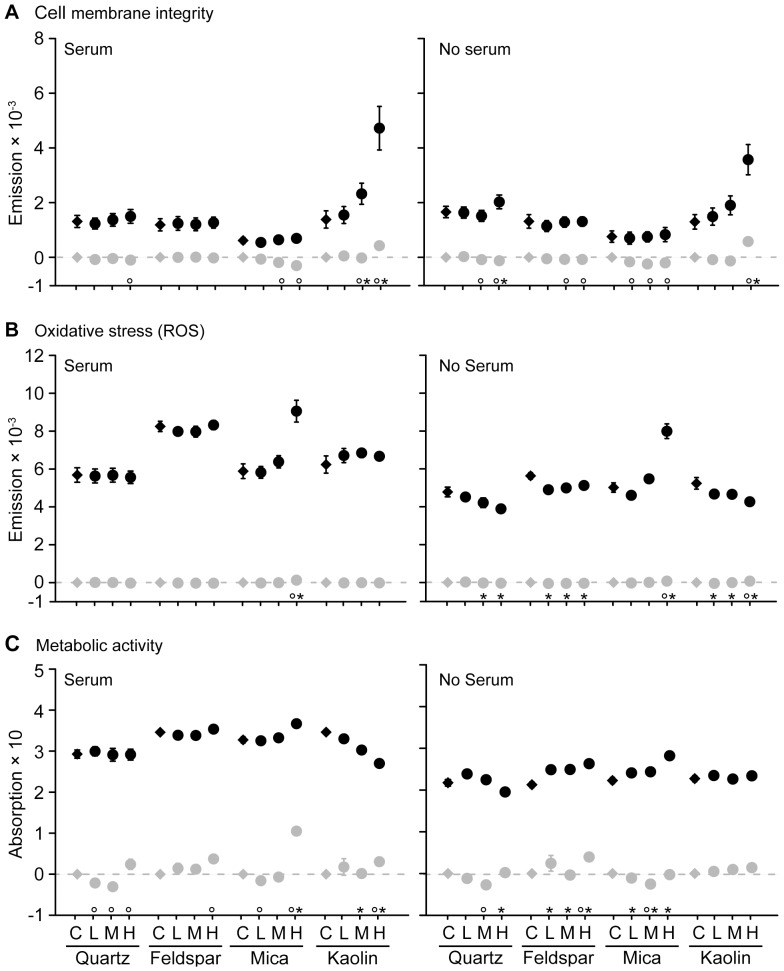
Cytotoxic effects and particle interference in the applied cytotoxicity assays. Shown are cytotoxic effects of the particles in cells (black symbols) and particle interference with the respective assay (grey symbols), all data after 72 h exposure. Symbols near the x-axis denote significant differences (p<0.05) to respective control for cytotoxicity (asterisks) and particle interference (circles) data. Labels on x-axis denote control (C, no particles), as well as low (L, 10 mg L^−1^), medium (M, 50 mg L^−1^) and high (H, 250 mg L^−1^) treatment level, grouped according to mineral species. Data points are mean ± SE. Note: Shown are summary statistics calculated from raw-data, while significance was tested with linear mixed-effect models to adjust for clustering of the wells on plates.

Particle interference with assays – All mineral particles had minimal effects on cell membrane intergity (PI) and on oxidative stress (H_2_-DCF-DA) (Figure 2A–C). However, the metabolic activity assay (MTT) was markedly affected by all particles, and the induced changes regularly exceeded the values observed in cell assays (Figure 2C). This was most prominent with 250 mg L^−1^ mica particles suspended in medium with FBS (Figure 2C).

Cell membrane integrity – At any concentration and time-point tested, the quartz, feldspar and mica particles had only minimal effects (≤1.1-fold change compared to control) on membrane integrity (Figures 2A, S1 and S2). In contrast, kaolin impaired cell membrane integrity markedly (1.2–1.6-fold change compared to control) at concentrations of 250 mg L^−1^, indicating a damaging effect of these particles on the cell membrane. This response pattern was observed at all time-points and independent of FBS presence in the medium (Figures S1 *vs* S2).

Oxidative stress – In this assay, the quartz, feldspar, and kaolin particles caused between 0.8 and 1.2-fold change compared to controls, indicating a slight effect of these particles on free radical levels (Figures 2B, S1 and S2). In contrast, the highest mica particle concentration (250 mg L^−1^) caused consistent increases of free radical levels at all time-points, as reflected in 1.3–1.8-fold changes with 10% FBS present in the medium (Figures 2B and S1) and 1.4–1.5-fold changes (Figure 2B and S2) without 10% FBS. Thus, mica particles caused a general increase of free radicals independent of FBS presence (Figures S1 *vs* S2).

Metabolic activity – The quartz particles caused a slight increase (≤1.2-fold change compared to control) in metabolic activity when cells were exposed ≤48 h in the presence of FBS. Values returned to control levels at 72 h and 96 h (Figures 2C and S1). Without FBS, quartz caused a slight decrease (≤1.1-fold change compared to control) in metabolic activity, mostly at the highest particle concentration (Figures 2C and S2). Feldspar caused no effects in cells cultured with FBS (Figures 2C and S1), but without FBS the metabolic activity was slightly increased (≤1.25-fold change compared to control), which was most pronounced at 72 h (Figure 2C and S2). For the mica particles the MTT assay indicated a slight increase (≤1.3- fold change compared to control) in metabolic activity of cells exposed to FBS (Figure 2C and S1). Likewise, in cells without FBS, 250 mg L^−1^ mica particles caused up to a 1.4-fold increase in metabolic activity (Figures 2C, S1 and S2). The kaolin particles caused a concentration-dependent 0.75–0.60-fold decrease in metabolic activity compared to controls in cells exposed for ≥72 h to FBS (Figures 2C and S1); without FBS no such marked decrease was observed (Figures 2C and S2).

### Relative cytotoxicity

Comparing the relative cytotoxicity (Table 2) revealed that quartz, feldspar, and mica hardly affected cell membrane integrity, while kaolin caused strong effects. In the oxidative stress assay, quartz and feldspar caused occasional slight effects, regardless of exposure duration and presence of FBS in the medium. Mica caused consistent moderate to strong increases in free radicals, regardless of exposure duration and FBS presence. With FBS in the medium, kaolin caused slightly increased free radicals in cells exposed for ≤72 h, which reversed to a slight decrease at 96 h. Without FBS, kaolin induced a slight decrease at all time-points ≤72 h.

**Table 2:** Relative cytotoxicity of the mineral particles.

FBS	Time	Quartz		Feldspar		Mica		Kaolin	
		PI	ROS	PI	ROS	PI	ROS	PI	ROS
Yes	24 h		-				++	+	+
	48 h		+				+++	+++	+
	72 h						++	+++	+
	96 h						++	+++	-
No	24 h						++	+	-
	48 h						++	++	-
	72 h						++	+++	-
	96 h						++	+++	

Given are ranked effect strength in the cell membrane integrity (PI) and oxidative stress (ROS) assays. Ranked effect strength was inferred relative to the maximum fold-change in the 250 mg L^−1^ particle concentration (Figures S1 and S2). Symbols denote response intensity as slight (+/-), moderate (++/—) or strong (+++/---). Empty cells denote no effect beyond ±10% from control. Fold-change intervals linked to the respective response intensities given in Table S1.

### Cell numbers

**Figure 3:**
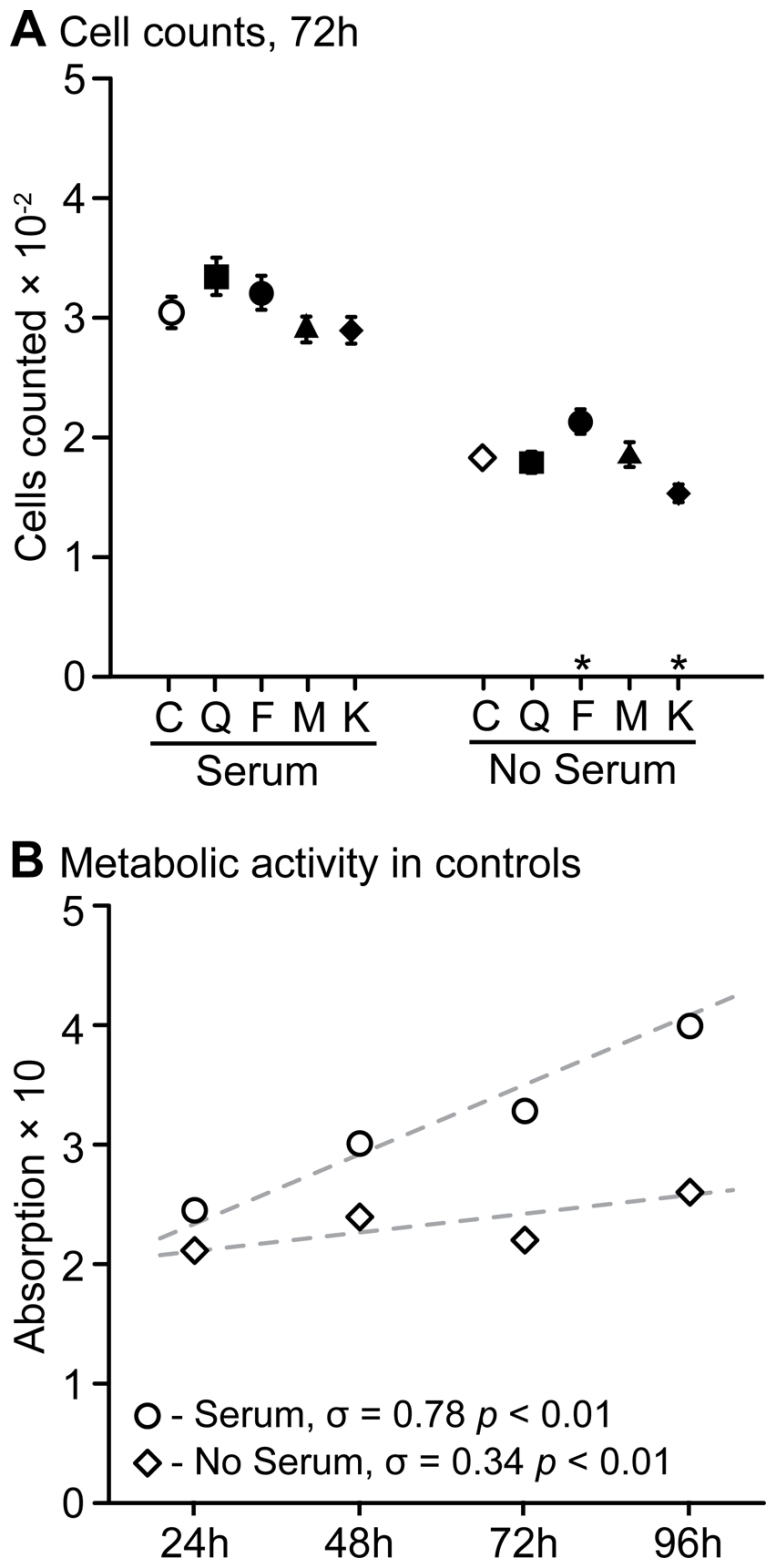
Effects in cell numbers and baseline metabolic activity in control wells. A.) Cell counts after 72 h exposure to 250 mg L^−1^ mineral particles. Shown are exposure with (left group) and without (right group) FBS. Open symbols are controls (C, no particles), and filled symbols denote mineral particle (quartz, Q; feldspar, F; mica, M; kaolin, K). Asterisks near the x-axis denote significant (p<0.05) differences to respective control; B.) Changes in metabolic activity of controls during the experiment, shown are controls with (circles) and without (diamonds) FBS. Dashed lines are best fit lines with respective Pearson product-moment correlation coefficients (σ) given near the x-axis. In both graphs data points are mean ± SE. Note: Summary statistics shown in A.) were calculated from raw-data, while significance was tested with a generalized linear mixed-effect model to adjust for clustering of the wells on plates.

After 72 h particle exposure, cell numbers in the presence of FBS were higher than for cells cultured in medium without FBS (Figure 3A). The highest particle concentration (250 mg L^−1^) had no marked effect on cell numbers after 72 h exposure (Figure 3A). Only for cells exposed without FBS, feldspar caused a slight increase and kaolin a slight decrease in cell numbers (Figure 3A). The metabolic activity confirmed the general increase in viable cell numbers in controls with versus without FBS (Figure 3B). In addition, it demonstrated that the numbers of viable cells increased over the exposure period, and that this increase was two-fold stronger for cells in the presence of FBS (Figure 3B). The difference in viable cell numbers was minimal at 24 h and peaked at 96 h, when a two-fold increase occurred in cells cultured with FBS (Figure 3B).

## Discussion

The current study provides first evidence for distinct effects of different natural mineral particles regarding cytotoxicity and uptake in salmonid gill epithelial cells. These data will be also useful for comparing the cytotoxic potential of natural mineral particles (NMPs) to that of manufactured nanoparticles (ENPs). Finally, our results indicate that natural mineral particles interfere with common cytotoxicity assays.

### Particle uptake and cellular effects

Regardless of their mineralogy, particles between 0.1 and 2 µm diameter were regularly internalized in RTgill-W1 cells. Most mineral particles were observed in membrane-bound vesicles, indicating that their entry into cells took place via phagocytosis (cf. [63], [64]). In the experiment the particles also settled on the cells, and the evidence for particle uptake presented here clearly suggests that the mineral particles came into contact and interacted with the cells. This conclusion is supported by ENP agglomerate uptake in RTgill-W1 cells [39]. Moreover, particles similar in size to the mineral particles investigated here are phagocytized in bird and mammalian respiratory epithelium cells *in vitro* [63], [65], [66]. Additionally, particle uptake in gill epithelia *in vivo* has been demonstrated in the blue mussel (ferric iron and plastic particles;[67], [68]) and in juvenile Pacific salmon (mineral particles; [45], [69]). Altogether, these data suggest that mineral particle uptake by gill epithelial cells is common in aquatic organisms.

The results presented here do not allow for quantification of the number of particles incorporated by the RTgill-W1 cells differed between the mineral species. Such differences have been demonstrated for ENPs of different composition [70] and also for differently sized albumin-coated microspheres (0.5–3 µm; [63]). Nonetheless, our data indicate that three out of four mineral particles did not cause visible ultrastructural changes in the RTgill-W1 cells. Only mica slightly dilated the endoplasmic reticulum. This effect was previously observed in cells experiencing free radical stress [71], [72]. The ultrastructural investigations also documented that, regardless of the cytotoxic effects triggered by certain particles, none of them caused pronounced cell disintegration or death. This agrees with the finding that particle exposure only minimally changed cell numbers.

### Mineral species affect cytotoxicity

The results presented here demonstrate remarkable differences in the cytotoxicity of four common fluvial silicate minerals in a rainbow trout gill cell line: feldspar was least cytotoxic, while the clay particles (mica, kaolin) were distinctly more cytotoxic than the framework silicates (quartz, feldspar). The generally increased cytotoxicity of the clay particles could reflect their smaller particle sizes and higher specific surface area [32]. In human lung epithelial cells and alveolar macrophages, stronger cellular effects occurred for mineral particle samples with higher specific surface areas [34], [73]. Cell numbers were hardly affected by the NMP exposure, and hence cytotoxic responses are most likely not related to changes in cell numbers. Accordingly, the results presented here indicate that natural minerals can cause cytotoxic effects in gill epithelial cells, with clay particles having an especially strong cytotoxic potential.

In addition to the generally increased cytotoxicity of the clay particles, the cytotoxicity assays also demonstrate that kaolin and mica affected the cells differently: kaolin caused cell membrane damage at concentrations of 50 mg L^−1^ (at 48 h and 72 h) and 250 mg L^−1^ (all time-points). This could also explain why only kaolin particles were occasionally found without a surrounding membrane in the cytoplasm. Evidence for cell membrane damage was weak for the mica particles. Instead, the highest mica particle concentration (250 mg L^−1^) induced consistent free radical stress. As pointed out above, this also caused endoplasmic reticulum stress. Two considerations make it unlikely that these differences in cellular effects between mica and kaolin were solely related to differences in the specific surface area: First, the particles caused markedly different cytotoxic effects, either by impairing the cell membrane (kaolin) or related to energy metabolism (mica). Second, mica had a two-fold higher specific surface compared to kaolin, indicating a two-fold higher surface area dose of the mica particles. A two-fold difference could not explain the cytotoxicological differences either in cell membrane integrity or in the oxidative stress assay. These data suggest that, like for other mineral particles in the low µm size range, additional contributing factors have to be assumed [32]. This agrees with studies in lung epithelial cells investigating mineral particles of different geochemical composition [34] or manufactured nano- and micron-sized particles [74], [75]. Among the most important factors that could have contributed are different functional surface groups [32], [33]. For fluvial suspended particles, biofilm and toxic substances bound to the particle surface could play a role [76]. Finally, shape differences could have contributed to the increased cell membrane damage caused by the kaolin particles [77]. An extensive study of all potential contributing factors was beyond the scope of the current experiments. Nonetheless, the results indicate that geochemical composition and possibly related surface properties affect the cytotoxic potential of clay particles in gill epithelial cells of aquatic organisms.

### Comparison with manufactured nanoparticles

Similar to the clay particles studied here, some engineered nanoparticles (ENPs) are known to cause cytotoxic effects in RTgill-W1 cells *in vitro*, and their effect also varied between particle types. For example, 25 mg L^−1^ palladium-magnetite ENPs did not affect cell viability after 72 h exposure [38]. When exposed for the same period, cobalt-doped tungsten carbide ENPs (30 mg L^−1^) decreased membrane integrity and metabolic activity [39]. Similarly, 45 mg L^−1^ gold ENPs decreased the metabolic activity in RTgill-W1 cells after 24 h exposure [40]. In an *in vivo* experiment, TiO_2_ ENPs caused lipid peroxidation in the gills of rainbow trout (1 mg L^−1^, 14 days exposure; [78]). Likewise, waterborne exposure to suspended fine sediment pulses (200 mg L^−1^ maximum concentration, 120 days exposure; [79]) and silver ENPs (0.1 mg L^−1^, 10 days exposure; [80]) induced the expression of genes in salmonid gills involved in protecting the tissue from oxidative damage. Recent research documented that suspended mica clay pulses induced slight lipid peroxidation in the gill of rainbow trout (300 mg L^−1^ maximum concentration, 24 days exposure; [16]). These *in vivo* results agree with the notion that oxidative stress can be a toxic effect of both NMPs and ENPs in cells [28], [30], [81]. However, our results also indicate that this effect pathway might not be triggered by all natural mineral particles but rather depend on specific particle properties, which remain to be investigated.

The results indicate that natural clay particles can induce cytotoxic effects comparable to manufactured ENPs in gill epithelial cells. For eco-toxicological risk assessment it would be important to know, firstly, whether ENPs are more cytotoxic than natural clay particles, and, secondly, if effect pathways differ between the two [21]. Question two cannot be addressed with the results presented here. For question one, however, the results, together with the research summarized in the previous paragraph, suggest that natural clay particles might be less cytotoxic: In the above-cited *in vitro* studies, the concentrations of ENPs causing cytotoxicity were consistently lower compared to the current study. Also, natural clay particles [13], [16], [17], [82] induced less structural damage in fish gills than some ENPs [78], [83]. The stronger cytotoxic potential of ENPs could be related to their higher specific surface area (e.g. 57–188 m^2^ g^−1^; [84]), but also novel surface properties and hence different surface reactivity [29]. This calls for quantifying the relative cytotoxicity of ENPs versus natural clay particles with similar specific surface area and/or particle sizes. Such information would also help understand if the tailor-made surface modifications of certain ENPs induce stronger or different cytotoxic effects – an important aspect for eco-toxicological risk assessment of ENPs [21]. Moreover, it also remains to be clarified to what extent the results of the numerous *in vitro* studies, including this study, can be transferred to conditions encountered *in vivo* and in fluvial ecosystems. All these data would clearly improve eco-toxicological risk assessments of both natural mineral particles and manufactured nanoparticles.

### Methodological implications

FBS addition had some effects on the responses observed in the cell assays. FBS can coat particles, affecting their surface properties and agglomeration, which could alter the interaction with cells and probably their cytotoxic potential (e.g., [39], [49]). Yet the data presented here provide no indication that adding FBS affected particle uptake and/or strongly altered the cytotoxic responses in the cell membrane integrity (PI) and oxidative stress (H_2_DCF-DA) assays. FBS did, however, have a marked effect on cell numbers in wells and their metabolic status. This has been previously demonstrated, also for the RTgill-W1 cell line [41], [85]. For indicator dyes that need to be metabolically activated by the cells (e.g. MTT, Alamar blue, H_2_DCF-DA) the number of viable cells and their metabolic status can affect assay results. In support of this notion, the PI assay, where the indicator dye binds only to nuclear DNA, was least affected by FBS addition. In the current experiment, FBS addition increased the number of viable cells; this was paralleled by an increased metabolic activity in controls at the latest after 48 h exposure. Therefore, studies on particle effects in cell culture experiments at time-points beyond 24 h, or even earlier in faster proliferating cells, should complement cytotoxicity data with an assessment of cell numbers.

The results further demonstrate that mineral particles interfere with cytotoxicity assays traditionally applied in particle toxicology research (e.g., [66], [86], [87]). Particle interference is a known problem in NP research, where marked interference occurred at concentrations as low as 10 and 30 mg L^−1^ (MTT and H_2_-DCF-DA assays; [55], [88], [89]). The mineral particles interfered mostly at concentrations ≥50 mg L^−1^. Effect strengths of the mineral particles were smaller than for ENPs [55]. This might reflect the smaller particle size of ENPs, which increases their specific surface area and particle reactivity [31]. Also, the washing step in the approach applied here likely reduced particle interference [55]. From these data it appears that mineral particles interfere less, at least in the cell integrity (PI) and oxidative stress (H_2_DCF-DA) assays. In the metabolic activity assay (MTT) the mineral particles caused sufficient interference to confound the interpretation of effects. Particularly with FBS, the highest mica particle concentration (250 mg L^−1^) altered the metabolic activity measured in cell assays. In light of these data, the MTT assay was least reliable for cell viability assessment after particle exposure. This calls for considering the interference of mineral particles with cytotoxicity assays in cell culture experiments. The particle interference controls applied in the current study, which enable to discriminate particle interference from particle effects in cells, are a minimum prerequisite to validate the results of such experiments.

## Supporting Information

Figure S1 **Cytotoxic effects in RTgill-W1 cells exposed in medium with 10% FBS.** Shown are effects on membrane permeability (PI assay), metabolic activity (MTT assay) and oxidative stress (ROS assay). Grouped data points in each graph represent exposure times (24, 48, 72 and 96 h), with symbols from left to right denoting control (open diamonds) and particle exposed cells (filled dots) in increasing particle concentration. Data points show mean ± SE. Asterisks above x-axis denote significant differences to respective control (p<0.05). Note: Data points show values calculated from raw-data, while significance was tested with linear mixed-effect models to adjust for clustering of wells on the plates.
(DOCX)

Figure S2 **Cytotoxic effects in RTgill-W1 cells exposed in medium without 10% FBS.** Shown are effects on membrane permeability (PI assay), metabolic activity (MTT assay) and oxidative stress (ROS assay). Grouped data points in each graph represent exposure times (24, 48, 72 and 96 h), with symbols from left to right denoting control (open diamonds) and particle exposed cells (filled dots) in increasing particle concentration. Data points show mean ± SE. Asterisks above x-axis denote significant differences to respective control (p<0.05). Note: Data points show values calculated from raw-data, while significance was tested with linear mixed-effect models to adjust for the clustering of wells on the plates.
(DOCX)

Table S1 **Fold change intervals for relative cytotoxicity ranking given in** **Table 1** **.**
(XLSX)
