# Supplementary material for: Natural Mineral Particles Are Cytotoxic to Rainbow Trout Gill Epithelial Cells In Vitro
Source: PLoS One. 2014 Jul 3;9(7):e100856. doi: 10.1371/journal.pone.0100856 (PMC4081506; doi:10.1371/journal.pone.0100856)
Supplement: Figure S2 — Cytotoxic effects in RTgill-W1 cells exposed in medium without 10% FBS. Shown are effects on membrane permeability (PI assay), metabolic activity (MTT assay) and oxidative stress (ROS assay). Grouped data points in each graph represent exposure times (24, 48, 72 and 96 h), with symbols from left to right denoting control (open diamonds) and particle exposed cells (filled dots) in increasing particle concentration. Data points show mean ± SE. Asterisks above x-axis denote significant differences to respective control (p<0.05). Note: Data points show values calculated from raw-data, while significance was tested with linear mixed-effect models to adjust for the clustering of wells on the plates. (DOCX) [file pone.0100856.s002.docx]

**

Figure S2 – Cytotoxic effects in RTgill-W1 cells exposed in medium without 10% FBS**.
